# OB3D, a new set of 3D objects available for research: a web-based study

**DOI:** 10.3389/fpsyg.2014.01062

**Published:** 2014-10-06

**Authors:** Stéphane Buffat, Véronique Chastres, Alain Bichot, Delphine Rider, Frédéric Benmussa, Jean Lorenceau

**Affiliations:** ^1^Département Action et Cognition en Situation Opérationnelle, Institut de Recherche Biomédicale des ArméesBrétigny, France; ^2^Cognition and Action Group, Cognac G, Service de Santé des Armées, Centre National de la Recherche Scientifique, Université Paris Descartes, Unités Mixtes de Recherche-MD 4 - 8257Paris, France; ^3^Centre National de la Recherche Scientifique, Unités Mixtes de Service Relais d'Information sur les Sciences de la Cognition 3332Paris, France; ^4^Laboratoire des Systèmes Perceptifs, Département d'études Cognitives, Unités Mixtes de Recherche-8248, Centre National de la Recherche Scientifique, École Normale SupérieureParis, France

**Keywords:** category, data-set, normalization, object denomination, web-based experiment

## Abstract

Studying object recognition is central to fundamental and clinical research on cognitive functions but suffers from the limitations of the available sets that cannot always be modified and adapted to meet the specific goals of each study. We here present a new set of 3D scans of real objects available on-line as ASCII files, OB3D. These files are lists of dots, each defined by a triplet of spatial coordinates and their normal that allow simple and highly versatile transformations and adaptations. We performed a web-based experiment to evaluate the minimal number of dots required for the denomination and categorization of these objects, thus providing a reference threshold. We further analyze several other variables derived from this data set, such as the correlations with object complexity. This new stimulus set, which was found to activate the Lower Occipital Complex (LOC) in another study, may be of interest for studies of cognitive functions in healthy participants and patients with cognitive impairments, including visual perception, language, memory, etc.

## Introduction

Sets of experimental visual stimuli are bread and butter for any research investigating cognitive functions in healthy individuals and patients. Continuous improvements of numerical editing and manipulation of images, as well as their dissemination through the Internet, helped the fast development and use of classes of visual stimuli coming in a variety of formats and representing a large diversity of “objects” whether natural or artificial. One such set comes from the seminal work of Snodgrass and Corwin (1988) who used 260 black-and-white line-drawings depicting objects, animals, vehicles, body parts, or symbolic representations, such as the sun or the moon. These pictures have been normalized through ratings of familiarity, visual complexity, or their matching level with the participants' mental representations. This initial set has subsequently been expanded and modified, and norms have been established for different language speaking communities (for a review see Brodeur et al., 2010), thus expanding the meta-data associated with these databases (Alario and Ferrand, 1999; Rossion and Pourtois, 2004). Recent modifications aimed at reducing stimulus information for testing specific processes related to visual recognition and object identification. For instance, De Winter and Wagemans (2004) used silhouettes, degraded, fragmented, and straight-line versions of pictures to evaluate the limits of contour-based integration and segmentation of nameable objects.

Other visual data sets are often made up from photographs transformed into different numerical formats. Efforts were made to provide well-controlled sets (Bonin et al., 2003; Rossion and Pourtois, 2004; Geusebroek et al., 2005; Brodeur et al., 2010; Jianxiong et al., 2010; Dan-Glauser and Scherer, 2011; Tkačik et al., 2011; Kovalenko et al., 2012; Moreno-Martínez and Montoro, 2012; Nishimoto et al., 2012; Umla-Runge et al., 2012; Migo et al., 2013). Although pictures of natural scenes and objects possess a number of advantages (colored and detailed representations, rich and complex environments), they also present limitations, mainly the fact that a single viewpoint is available and that these images are static. Adapting and transforming these stimuli to meet specific experimental requirements is difficult because specialized software is needed and editing is long and costly. Controlling and manipulating low-level characteristics or making animated versions is sometimes simply impossible.

**Figure 1:**
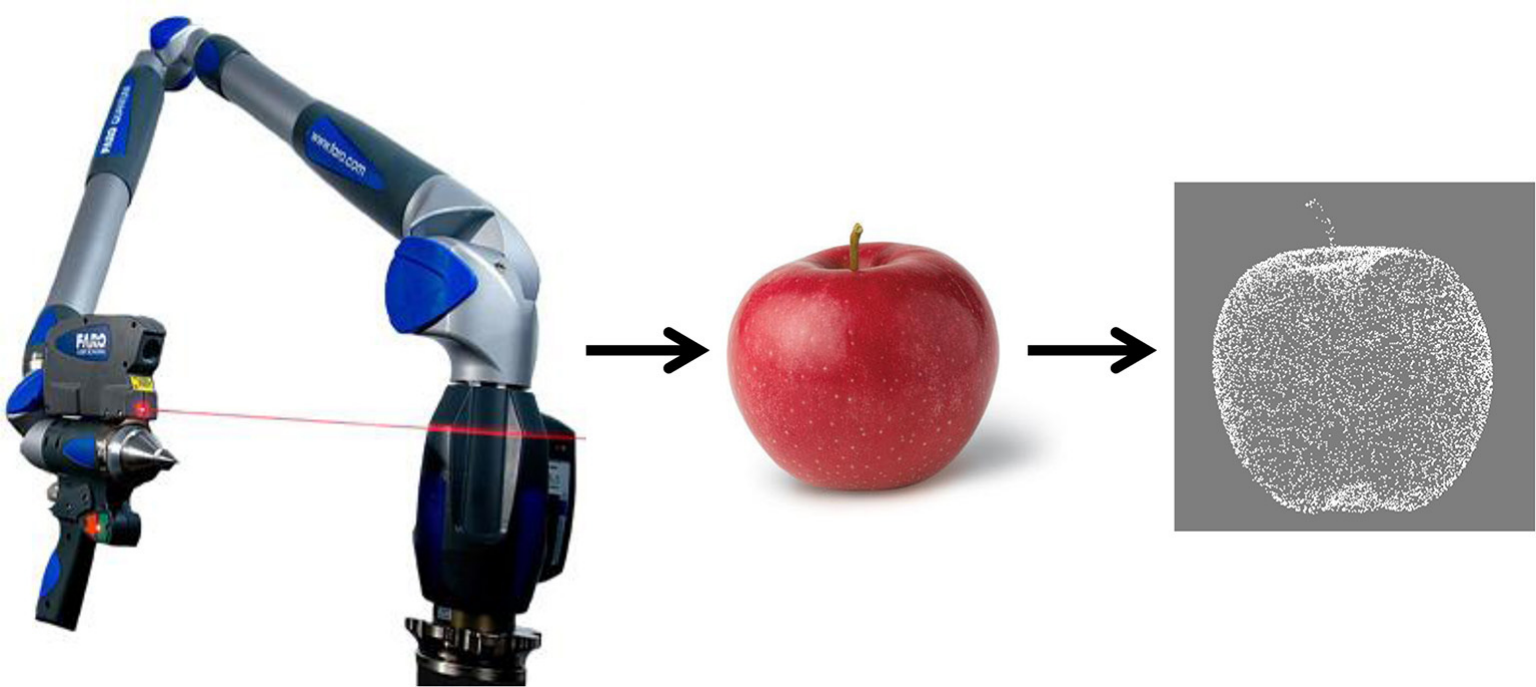
**Stimulus generation. Left: Faro laser scanner used to generate the object dot clouds. Middle:** Example of a real world object. **Right:** Cloud of dots representing the scanned object. Each dot is defined by X, Y, and Z coordinates as well as the normal to the surface. Each dot cloud defining one object is available in different formats as an ASCII file. The OB3D database is free, open source and can be downloaded online at http://ob3d.scicog.fr/.

We took a different, complementary, approach and designed a new set of ~140 visual stimuli, constructed by scanning real 3D objects (either “natural” or realistic toy versions of “natural” objects) with a laser scanner (Figure 1). In this way, we obtained lists of 3D coordinates of dots depicting these objects, available as “ASCII text files” that can easily be displayed, edited and modified. Different formats are available (^*^.x3d, ^*^.wrl) together with free software that can be used for visualization.

**Figure 2:**
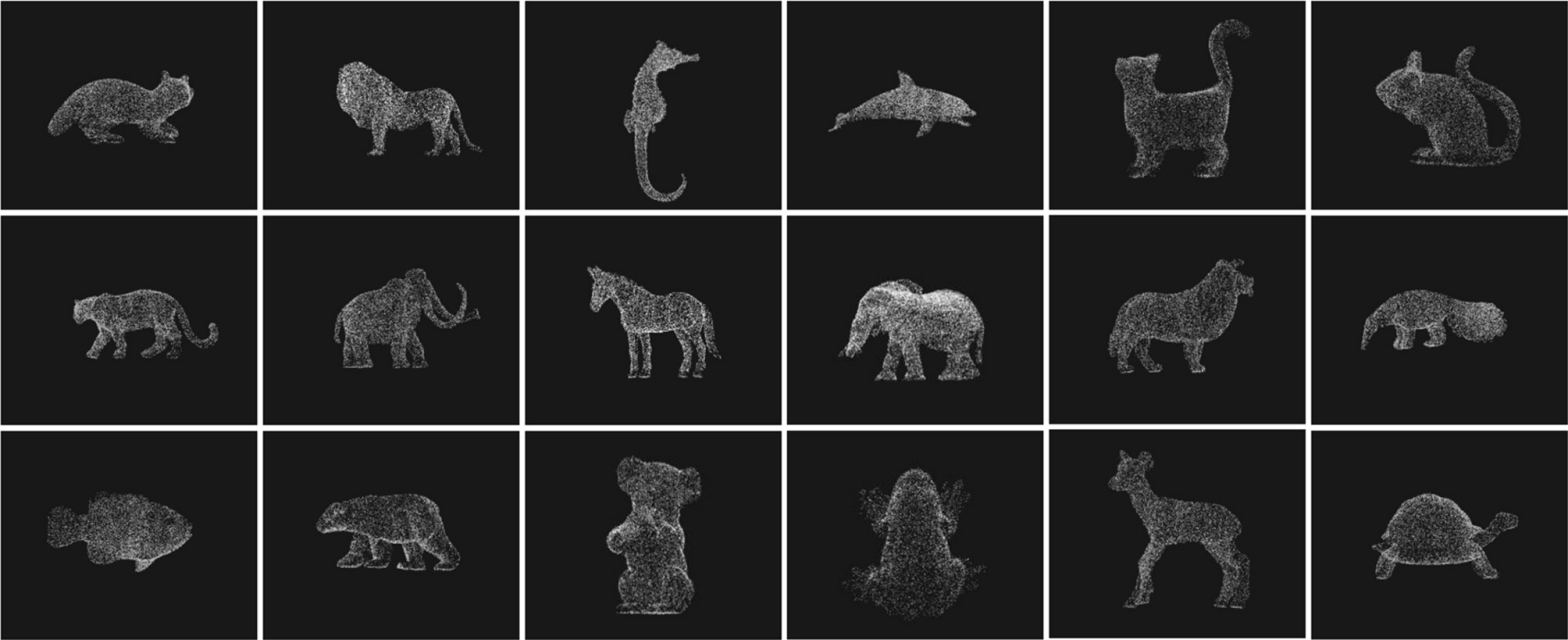
**Examples of 18 scanned objects from the OB3D set are presented using a subsample of 10,000 dots picked up randomly amongst all available dots**. In the web experiment, each 3D dot cloud was presented in isolation, starting with 100 dots whose number linearly increased until recognition.

With these stimuli, simple routines permit versatile transformations that can be performed in real time (see Figure 2). There are, however, limitations to this approach: the rendering of objects, in its simplest format, is not realistic as it lacks contour, color and texture, as well as diagnostic features that often provide a key to object recognition. The appearance is that of a transparent silhouette made of dots. Although it is in principle possible to overcome these limitations by further editing the stimuli with dedicated software, we leave this possibility to future work.

This stimulus set has been used in fMRI imaging studies that uncovered brain regions overlapping those already found to respond to objects (e.g., in the Lateral Occipital Cortex or LOC; Kourtzi and Kanwisher, 2001). MEG recordings further revealed temporal object related activations in the temporal lobe (Benmussa et al., 2012).

This stimulus set must be normalized to ensure that objects are consistently recognized across observers and associated meta-data should be made available to a large community. To that aim it is necessary to collect a large amount of data with numerous participants. In this regard, a web-based protocol has the advantages of easily and quickly collecting numerous answers via the world wide web, (Birnbaum, 2004). In addition, data collection can be done 24 h a day, and 7 days a week. Because experimental procedures are automated, the cost and the amount of time spent managing the experiment is reduced (Reips, 2000). The first experiments done in this way can be traced back to 1996 (Welsch and Krantz, 1996; Musch and Reips, 2000). There are however known issues, and the specificities of web-based experiments must be taken into account to analyze the results. One drawback is that web-based studies have a larger dropout rate than lab studies. Participants can simply abandon the on-going study outside a direct supervision, feeling neither social pressure nor embarrassment to do so (Frick et al., 1999; Knapp and Heidingsfelder, 2001; O'Neil and Penrod, 2001; Birnbaum, 2004). The second issue is that participants running online experiments are usually diverse and mostly unknown. In addition, the environmental conditions, such as lighting, display characteristics, ambient sounds, are vastly disparate and cannot be easily controlled for, and response biases induced by the design of the response page, or by subtle cues given to participants can occur. Sometimes, combining a web-based experiment with a laboratory experiment permits to control for some of these biases (Dandurand et al., 2008).

The aim of the present work is: (i) to advertise the stimuli stored in the OD3D available database and to present the results of the normative tests conducted with this set similar to Snodgrass and Vanderwart's (1980); (ii) to measure the minimal number of dots (dot threshold) needed to recognize, categorize and identify the OB3D objects which provide a quantitative measure of the minimum information needed to recognize and categorize these 3D objects.

## Materials and methods

### Participants

The experiment was promoted through mailing to the RISC (“Relais d'information sur les sciences de la cognition,” www.risc.cnrs.fr) volunteers' database (http://expesciences.risc.cnrs.fr/contenu.php).

Participants ranged between 22 and 54 years old (Mean age 28.6 years, ±7.5; women/men ratio of 63:37), and were native French speakers. All reported having no neurological disease and having normal or corrected to normal vision. The experiment was done by visiting the experiment's website (http://cogitolabo.risc.cnrs.fr/ob3d.php) and could be performed in different sessions.

Participants gave their consent before starting the experiment and were explained that they were free to stop at any time and for any reason. If they stopped early, participants received a password to reconnect to the web site and to continue the experiment where they left it, if they wished.

In total, 430 connections were registered, corresponding to a total of 223 different participants. Two participants who gave responses unrelated with the task and 11 participants who responded too quickly, resulting in mostly empty files recorded, were excluded from the analyses. In total, we analyzed the data from 210 participants.

### Stimuli

3D Objects from the OB3D database are free, open source available online (http://ob3d.scicog.fr/). The only requirement is to cite the website and to provide feedback such as data, links to articles or to new objects, etc. The 3D objects were created by scanning real life objects or “toy objects” with a Scan arm® Faro laser scanner (http://www.faro.com) allowing fast and accurate object acquisition. This hand-held laser scanner creates a 3D image through a triangulation algorithm: a laser line projected onto an object is reflected on a sensor measuring the distance to the surface, using an internal coordinate system provided by calibrated internal sensors. The scanned object lay on a flat plane known to the system such that extra points belonging to this plane are removed. Scans from different viewpoints are assembled using manually defined homologous points from different views. The 3D clouds of X, Y, Z ASCII coordinates of each object are available in several formats [.wrl,.obj,.wrp. See reference for.wrp (wraped file) with Geomagic software^tm^ http://www.geomagic.com/]. Further manipulations and transformations are fairly easy, because of the complete characterization of the object. Other formats are available (Polygons, vectors), but are more resource intensive. The whole procedure is described in Figure 1.

On average, 10^5^ points defined each object. Figure 2 presents down-sampled (10000 dots) versions of objects from the set.

**Figure 3:**
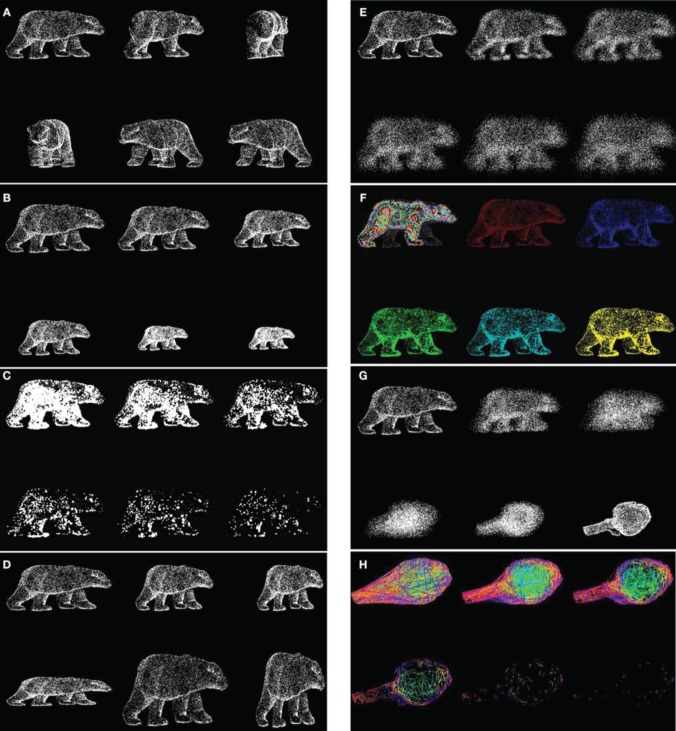
**Versatile transformations allowed by OB3D**. Each of these transformations can be rendered as a dynamic movie or as static snapshots. **(A)** Rotation of a “bear cloud” along the vertical axis, offering different 3D viewpoints. **(B)** Changing size. In these examples object size is modulated by changing the distance between dots. Expansion and contraction of 3D dot cloud. **(C)** Changing dot number. In these examples dot size is modulated by depth (*z* coordinates). **(D)** Varying the Vertical/horizontal aspect ratio of shapes. **(E)** Blurring by adding positional noise to 3D clouds coordinates. **(F)** Modulation of object appearance through color-coding. **(G)** Smooth morphing of one 3D cloud into another (morphing the distance between two homolog dots along each axis). **(H)** “Texturing” by connecting lines between neighboring dots. Color-coding is derived from the depth coordinates. Other modifications are possible: mixing 3D clouds and titrating the number of dots belonging to one or another object; deriving “scrambles” versions, etc.; editing OB3D objects with dedicated 3D software further permits realistic triangulation and texturing, lighting control or shadow rendering.

With these stimuli, a large number of object transformations are possible, such as rotating objects, decreasing the number of dots, changing the size, and proportions, adding positional noise, changing color, generating scramble versions, mixing, and morphing between objects, etc. (see Figure 3 and Supplementary Video 1).

XML files were used for the Web-based experiment. For each object, the point of view was manually set, so as to ease the recognition of each object, and the names and categories were assessed by means of the French lexical database “Lexique 2” (New et al., 2004; http://www.lexique.org/).

### Procedure

The web-based experiment unfolded as follows: After a blank page, 100 dots randomly picked up amongst the all the dots from a cloud object were presented. The number of dots then linearly increased with time. The participants were instructed to press a key when they were confident they had recognized the object. After a key press, the number of dots presented on the screen was recorded, and the response screen was displayed. At this point, participants had to fill out a short questionnaire: (1) To give the name of the object, or to answer “does not know the object” (DKO), “does not now the name” (DKN), or “tip of the tongue” (TOT); (2) To rate the object familiarity on a 0–9 scale; (3) To indicate the category of the object (forced choice). Afterwards, participants pressed a key to start the next trial, and a new object was presented. A random stimulus sequence was generated for each participant. Because each trial takes time to perform, after each 20 trials, participants could stop the experiment. In this case, they received a link by email allowing them to resume the experience later at the trial where they stop. The instructions are presented in Table 1.

**Table 1:** **Table summarizes the instruction given to the participants on the explanation page and the response pages on the central column**.

**Experiment-variables**	**Instructions**	**Methods**
Name	Identify the object as quickly and unambiguously as possible by writing only one name, the first name that comes to mind	Free text, or alternate checking box for DKO, DKN, and TOT answers^*^
Category	Indicate to which category the object belongs to	Forced choice (eight alternatives)
Familiarity	Rate the level of familiarity with the object	Rating scale 0–9
Retrospective confidence judgment	Rate your confidence to the naming question	Rating scale 0–9
Number of dots	Each object will be displayed with an increasing number of dots on the screen. Press a key as soon as you believed you recognized the object	Starts with 100 dots randomly picked up amongst all the dots. Linear increase of dot number until a Key is pressed
Free comment	Add any comment about the experiment or the object	Free text

*The actual method for answering is provided in the left column*.

* *DKO, don't know the object; DKN, don't know the name; TOT, Tip of the tongue*.

**Figure 4:**
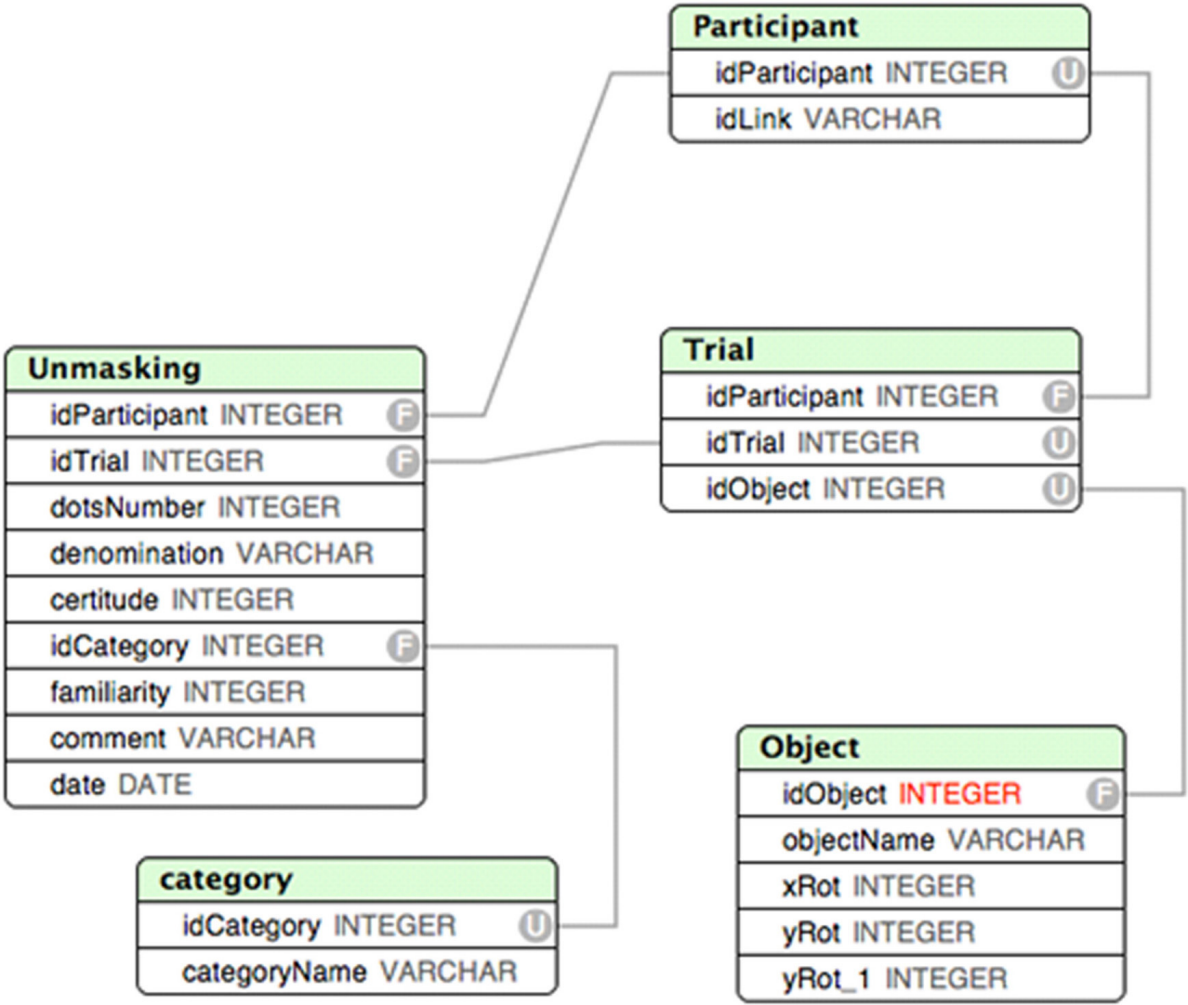
**Database software architecture**.

The web-based architecture was as follows:

The main functionalities of the experiment were written in HTML5 and JavaScript. The website was tested with all major web browsers and optimized desktop computers. Prior to the experiment proper, participants could test whether their browser supported WebGL (a JavaScript API for the rendering of interactive 3D graphics) and explained how to enable WebGL. All data were stored in a relational database, MySQL (number of dots needed to recognize the object, and the questionnaire answers). The database architecture is presented in Figure 4.
The experiment follows most of the recommendations of Birnbaum (2010), and complies with most of the standards for internet-based experiments provided by Reips (2002): First, the analyses were decided before designing the web-based experiment. Second, the programming of the study was checked for bugs several times, both off-line and online. Five participants pre-tested the experiment in the lab. The experiment started on July the 2nd, 2013 and lasted until July the 31st, 2013.

### Analysis

1. Ratings and correct answers
  Whenever the minimum number of displayed dots was 150 or less, the corresponding trial was excluded as it most likely corresponded to manipulation errors (no answer whatsoever for the questions). Only three such trials were discarded from further analyses, which accounts for a high rate of participants correctly performing the task.
2. The analysis mostly follows the same logic as in Brodeur et al. (2010). We computed the detailed descriptive statistics. The variability of the responses was studied by means of *H* values for naming agreement (*H*_name_) and categorization (*H*_cat_). The H statistics is sensitive to the number and weight of alternative names (or categories) and is computed as follows:
  $$H=\sum_{i\;=\;1}^{k}P_{i}Log_{2}\left(1/P_{i}\right)$$
  In this equation, *k* refers to the number of different names given to each picture. It excludes the DKN, DKO, and TOT responses because they do not provide alternate names for a given object. *P_i_* is the proportion of subjects who gave a name for each object. The *H* value of an object with a unique name and no alternative equals 0. The *H* value of an object with two names provided with an equivalent frequency is 1.00. The higher the number of alternate names, the higher *H*. The *H* statistics was computed for names (*H*_name_)and categories (*H*_cat_).
3. We also computed correlations (linear regression analysis) between correct answers and perceived familiarity, perceived difficulty, and the mean number of dots required for object recognition.
4. Free comments were coded, and the most frequent were analyzed. Most analyses are Chi^2^ We also computed *t*-test when appropriate.

## Results

**Figure 5:**
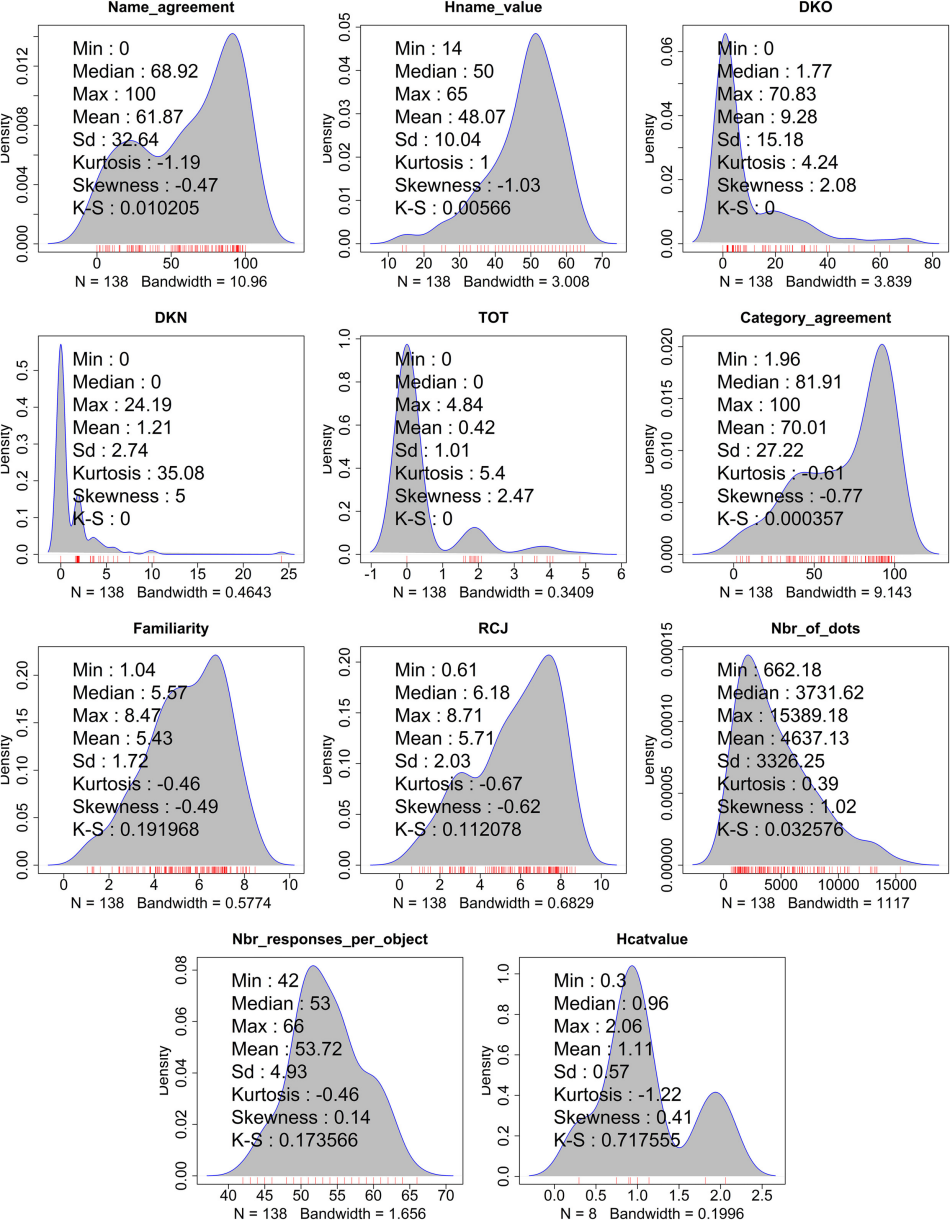
**Density distributions with rugs for all Norms**. The norms are labeled as follows: Name agreement, *H*_name_, DKO (Don't know the object), DKN (Don't know the name), TOT (Tip of the tongue), Category agreement, Familiarity, RCJ (Retrospective Confidence Judgment), Number of Dots, Number of Responses per object, and *H*_cat_. Note that in the case of DKO, DKN, and TOT, Min and Max value are computed as the min and max correct response percentages per object. All other Min and Max depict the range value for the possible answers. The following data are also displayed over the density distributions: Minimum (Min), Median, maximum (Max), Mean, standard deviation (SD), Kurtosis, Skewness, and a Kolmogorov–Smirnoff test (K–S).

In total, we collected 7200 answers, from 210 participants. The norms are summarized in Figure 5. All the norms presented are means across responses. All stimulus-specific norms are presented in Annex 1, to provide metadata for all the objects of the OB3D database. See also data for *H*_name_ and Name agreement plotted for each object in Supplementary Figure 1.

### Number of dots

**Figure 6:**
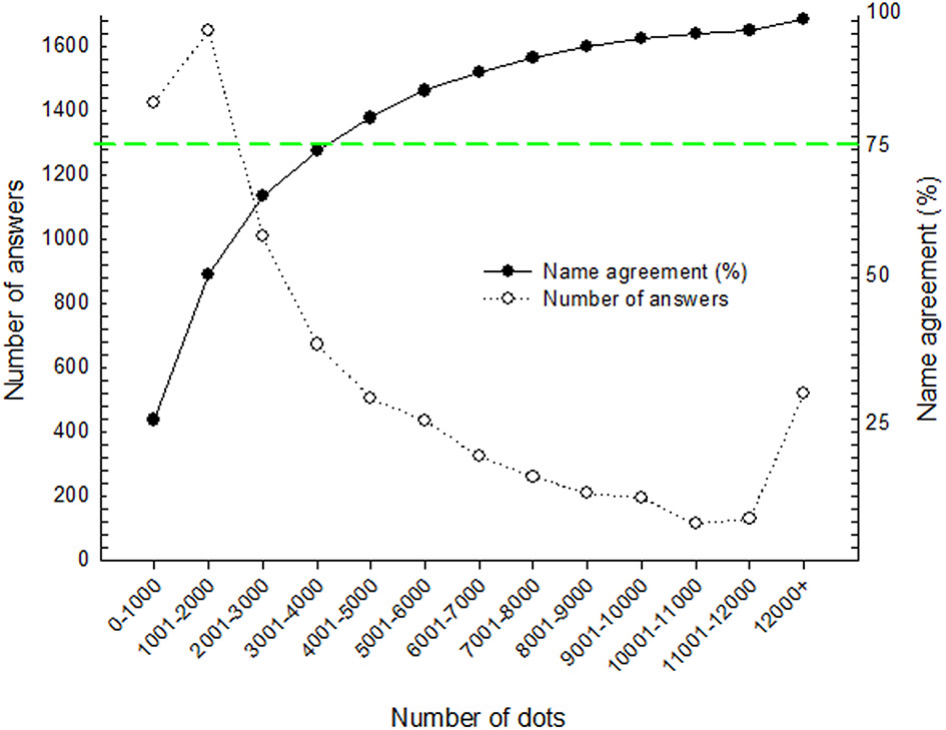
**The white dots represent the number of answers against the number of dots binned in 13 categories by steps of 1000 points**. The black dots represent mean correct responses per categories (Right scale). The (green) dashed line corresponds to 75% correct response rate for naming agreement.

The number of displayed dots allowing a correct naming provides a relevant quantitative indication of the minimum information needed to recognize and classify an object. The mean number of dots yielding correct recognition was 4560 (±6222), but varies widely, both across objects and observers, indicating varying degrees of ambiguity and uncertainty (see Supplementary Table 1). This variability possibly reflects misleading or irrelevant initial guessing when only a limited number of dots are available. As objects depicted by only few dots are compatible with a large set of possible objects, participants may need more dots to disambiguate the stimuli while other objects less prone to such “false priming” can be recognized more easily. In Figure 6, mean correct response rates are plotted against the number of displayed dots. We have binned these numbers in categories of 1000 points. Note that the mean number of dots for which participants reached 75% of correct responses for name agreement is comprised between 3001 and 4000.

### Names

Mean Name agreement was 62% (±48%). This result is very close to the results of Brodeur et al. (2010). Mean *H*_name_ was 1.69 (±0.11), also very close to the 1.65 (±1.10) found in the study of Brodeur et al. (2010). In both studies, the results indicate that the participants used more alternate names to identify the objects than in previous studies [e.g., *H*_name_ between 0.56 (±0.53) and 1.16 (±0.79) in Snodgrass and Vanderwart, 1980; Bates et al., 2003, respectively]. This discrepancy can mainly be attributed to the selection and number of the objects in each database. A large object database will contain objects that are more difficult to name than a small one. Depending on the intended use, it can be advantageous to have samples of objects that are more difficult to name, and others that are less difficult. This variability is especially important for clinical research with patients with cognitive impairments (Rizzo et al., 2000). That our stimuli appeared progressively as more dots were displayed, rather than being displayed at once, does not seem to have impaired name agreement. However, the viewpoints were fixed and had been chosen to maximize name agreement. In their paper, Brodeur et al. (2010) discuss the effect of color and details in name agreement. Our objects were presented in white dots over an uniform gray background. Thus, color did not participate in the visual recognition. However, the level of details is another matter. Although one can argue that a photograph is a highly detailed stimulus compared to a line drawing, our stimuli have fine details. Edges are less well defined in our stimuli, but the laser scanner very finely captures the structure details.

### Categorization

The mean for Category agreement was 70.2% (±4.58%). The categories with the highest mean for Category agreement were tools [84.3% (±3.64%)] and animals [82.6% (±3.80%)]. The categories with the lowest mean for Category agreement were furniture [50.0%, (±5.09%)] and others [21.8%, (±4.13%)]. With a mean *H*_cat_ of 1.10 (±0.57), our participants did not have many alternate categories. This result is somewhat to be expected with our forced choice procedure that included a category named “others.”

### DKN, DKO, and TOT

Mean DKN was 9.1% (±2.8%), mean DKO was 1.2% (±1.1%), and mean TOT was 0.4% (±0.6%). The sum of DKN and TOT is relatively high, which is consistent with the trade-off between having a large number of objects and easily named list of objects. This result is also in agreement with the Name agreement found in this experiment.

Contrary to previous studies, we did not offer more than eight categories. However, the number of objects in each category was almost the same, which gave our participants a balanced set of objects.

### Familiarity

The familiarity average ratings ranged over a scale from 0 to 9 (9 being very familiar). Mean Familiarity rate was 5.45 (±3.18), meaning participants were moderately or highly familiar with the objects. This is confirmed by performing a pairwise Welsch *t*-test between the familiarity ratings and the value 4.5, the middle point of our 10-point Likert Scale (*t* = 26.06, *p* < 0.0001, 95%inf. *CI* = 5.39; 95% sup. *CI* = 5.535). The familiarity is slightly lower than in previous studies (e.g., Brodeur et al., 2010). This difference could be due to the nature of our stimuli, made of dots. An alternate reason might be that 5-point scales, such as used in the literature, can skew the results toward the upper part of the scale (Preston and Colman, 2000).

### Retrospective confidence judgment

The Retrospective Confidence Judgment (RCJ) ratings ranged over a scale from 0 to 9 (9 being very Confident with participant's own response). Mean RCJ rate was 5.71 (±3.13). Overall, it indicates that participants were somewhat confident in their answers as confirmed by a pairwise Welsch *t*-test between the familiarity ratings and the value 4.5, the middle point of our 10-point Likert Scale (*t* = 34.34, *p* < 0.0001, 95%inf. *CI* = 5.67; 95% sup. *CI* = 5.82). This result, similar to that of Kennedy and Yorkston (2000) in healthy adults, gives a useful indication about the meta memory related to the objects presented in the experiment. This is especially relevant when one wishes to use such stimuli to test patients with brain injury, whether traumatic or following X-rays therapy (Kennedy, 2001).

### Correlations

**Figure 7:**
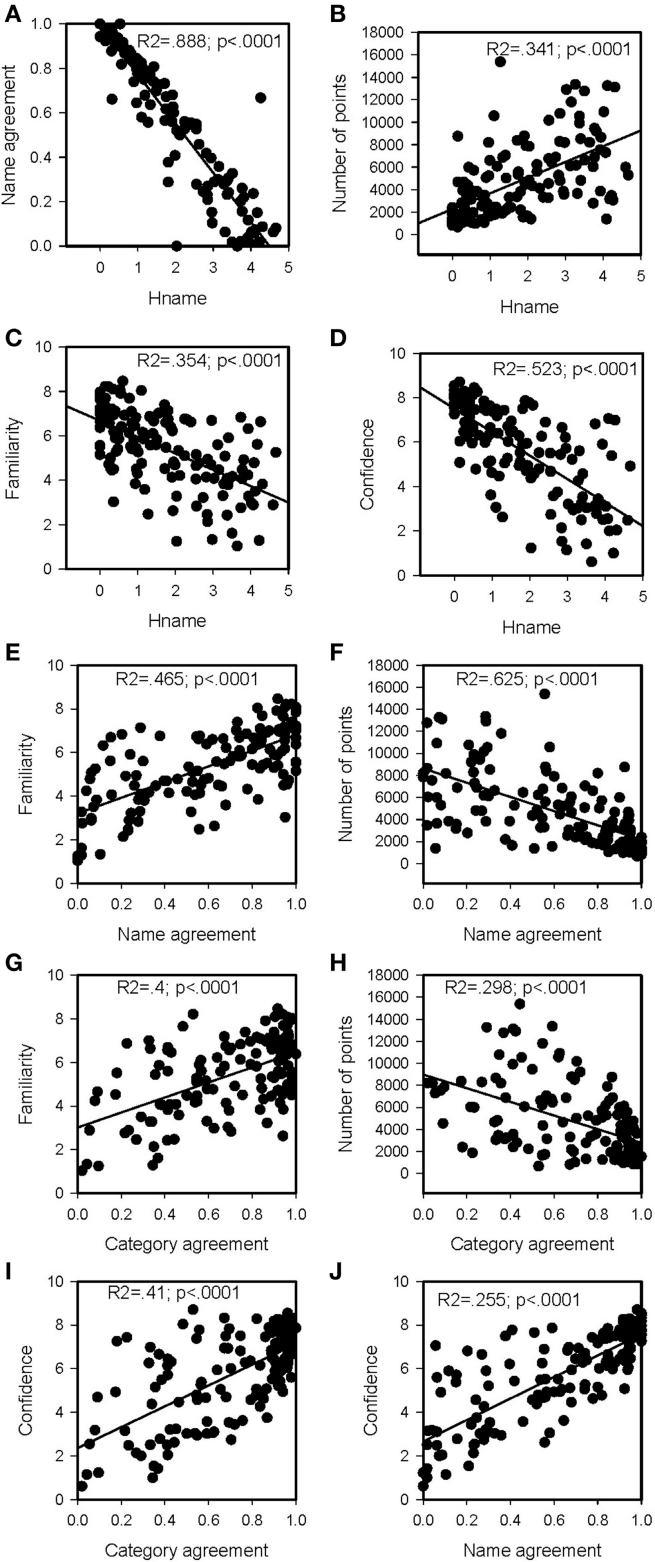
**Scatter plots of the correlations for *H*_name_, name, and category agreement. (A)** *H*_name_ × Name agreement; **(B)** *H*_name_ × Number of dots; **(C)** *H*_name_ × Familiarity; **(D)** *H*_name_ × Retrospective Confidence Judgment; **(E)** Name agreement × familiarity; **(F)** Name agreement × Retrospective Confidence Judgment; **(G)** Name agreement × Number of dots; **(H)** Category agreement × familiarity; **(I)** Category agreement × Retrospective Confidence Judgment; **(J)** Category agreement × Number of dots.

Correlations in normalization studies help understanding how different dimensions relate to each other. Table 2 presents the matrix of correlations, and Figure 7 presents the scatter plots of the correlations. The *H*_name_ gives an idea of the dispersion of the naming results. This result can be due to true alternate names, systematic errors, or uncertainty.

**Table 2:** **Matrix of correlations**.

**Variables 1**	**Variables 2**	**Sign**	***R*^2^**	***p***
*H*_name_	Name agreement	−	0.888	<0.0001
*H*_name_	Number of points	+	0.341	<0.0001
*H*_name_	Familiarity	−	0.354	<0.0001
*H*_name_	Retrospective confidence judgment	−	0.523	<0.0001
Name agreement	Familiarity	+	0.465	<0.0001
Name agreement	Retrospective confidence judgment	+	0.625	<0.0001
Name agreement	Number of points	−	0.4	<0.0001
Category agreement	Familiarity	+	0.298	<0.0001
Category agreement	Retrospective confidence judgment	+	0.41	<0.0001
Category agreement	Number of points	−	0.255	<0.0001

*Note that we did not calculate correlations with H_cat_ because of the forced choice paradigm (see text for a detailed explanation)*.

Most previous studies have shown that modal name agreement and the *H* value are negatively correlated. In addition, correlation between modal name agreement and the H value, as reported in the literature, are the strongly correlated variables with line-drawn pictures. The 0.888 is close to the 0.900 reported in the literature (Brodeur et al., 2010).

The correlation between *H*_name_ and Familiarity is also close to the 0.400 reported in Brodeur et al. (2010).

RCJ is positively correlated with name agreement: this relationship between accuracy and RCJ is consistent with the consensuality principle (Koriat, 2008). The negative correlation between RCJ and *H*_name_ found here is also an indication for this behavior.

### Free comments

The participants made 712 free comments, over a total of 7434 answers (9.7%). Overall, this is a good indication that the task was performed without any major issue for the participants. These comments were broke down by means of coding (see Table 3).

**Table 3:** **Coding of the free comments**.

**Coding**	**Locus**	**Meaning of the coding**	**Verbatim**
No comment	None	The space was left blank	
Explicitly no comment	None	The participant wrote an indication that she/he had no additional comment	“No”
Personal comment	Internal	Some piece of humor, something personal	“I am a veterinary”
Technical issue	External	There was a technical issue that could have impaired the experiment	“There was a brief black screen”
Perspective/viewpoint	External	The way the object was depicted in 3D seemed unusual	“The viewpoint is weird for this object”
Task difficulty	External	The task was thought to be too difficult	“This is too hard”
Alternate name	Internal	The participant proposed another name	Answer is “Plane,” comment is “Dart”
Sentence	Internal	The participant made a whole sentence to describe the object	“It would have been a mango if smoother”
Confidence	Internal	The participant expressed a certain level of confidence	“I am unsure”
Justification	Internal	The participant justified her/his choice	“The shoe has high heels”
Change with time	Internal	The participant stated that she/he would have initially answered a name, and then changed the Name at some point during the trial	“I first took if for a tree. I was right to wait for it was a vehicle”

**Figure 8:**
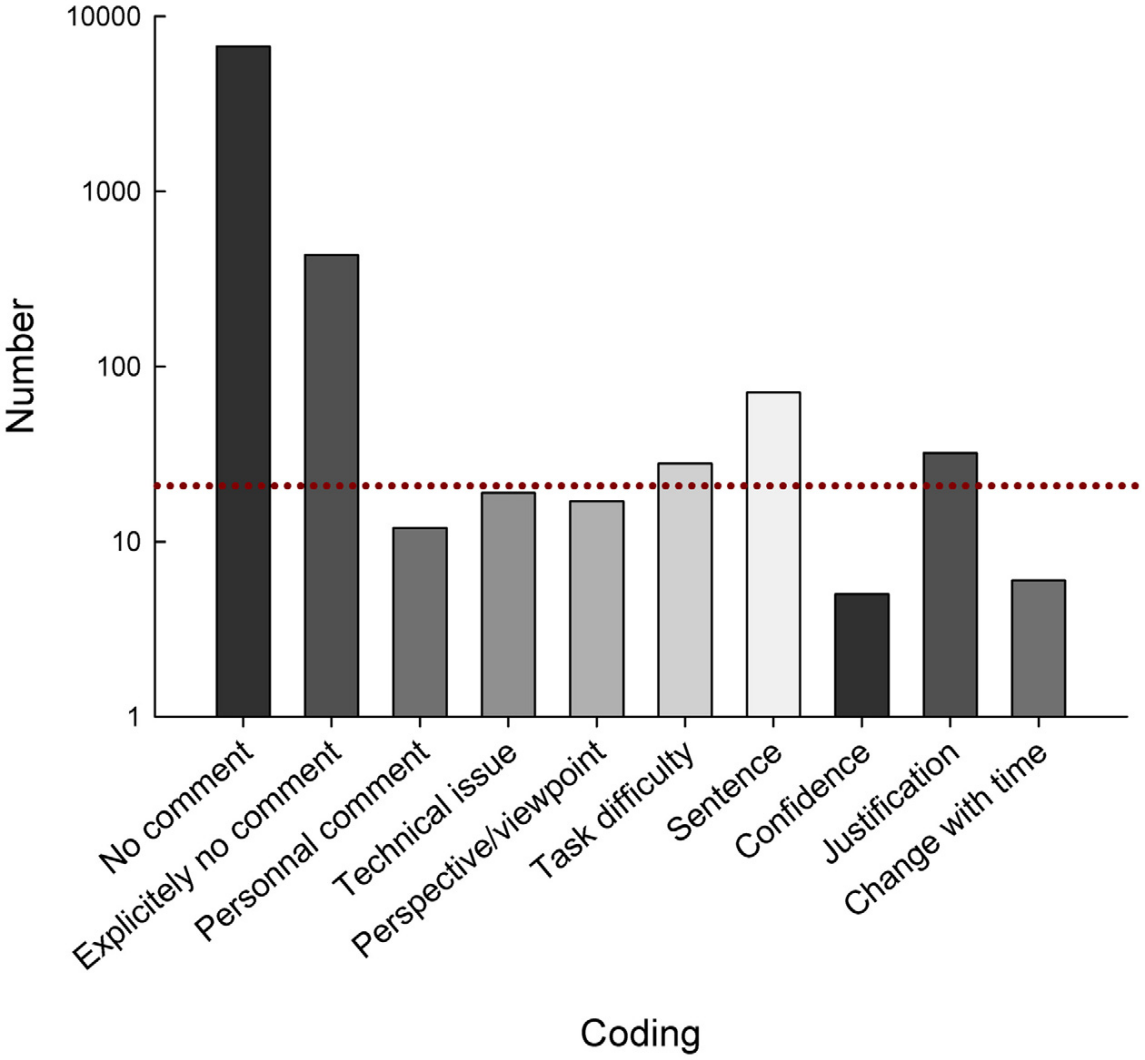
**Bar chart depicting the number for each coding of the free comment**. Note that the Y axis is a Log scale. The dotted line represents the arbitrary threshold we used to determine which comments we would address in the analysis.

The number of each coded comment is displayed in Figure 8.

We performed additional analyses for the three most common comments (more than 20), “Alternate name,” “Sentence,” and “Justification.”

When the participants made a comment coded “Alternate name,” they were more often wrong when naming the object (Corrected Chi^2^ = 0.044; Corrected *p* = 0.0002).

We performed a Chi^2^ analysis between the answers coded “Sentence” and those coded otherwise, regarding the TOT variable. There is a significant difference between the two cases, in favor of the participants expressing whole sentences to try to explain the object they saw, but being unable to name it correctly. Because there are few TOT, we used Fischer's exact probability (Corrected Chi^2^ = 16.63; Fischer's Exact Prob. = 0.0031).

When considering the answers with “Justification,” we found that they were not different than without in terms of name agreement (Corrected Chi^2^ = 0.045; Corrected *p* = 0.8553). However, participants reported being more familiar with the object (*t* = 2.16; *p* = 0.0308) and were more confident in their answers (*t* = 2.7; *p* = 0.0069) when such comment was present than when it was not.

We also pooled the comments in 3 Loci, “None,” “Internal,” and “External.” This gives some insight in the locus of control of the participants that made free comments. We found that 67% of the comments can be attributed to the internal locus, the remaining 33% being related to the external locus.

## Discussion

The present work describes a web-based experiment aimed at the normalization of a novel visual stimuli data set. This experiment was done in order to illustrate both the stimuli properties and on how valuable a web-based can be regarding database normalization. The OB3D is a free database of 3D objects that can be used by themselves, or embedded in virtual reality (VR) settings, with a comprehensive normalization. This data set is the first of its kind because one can easily customize the stimuli to fit with the experimental paradigm chosen by the researcher or clinician, and still be controlled for low-level vision cues.

### Normalization and controllability

The normalization results include RCJ in addition to the more widely reported parameters. First, we found normalization data consistent with the literature. Second, we provided additional value by providing a threshold in terms of the numbers of dots required reaching certain recognition rates. We believe that controllability of a stimulus is of paramount importance for neuropsychology tests. Other issues may arise, such as the necessity of control responses in a reference population (Rowe and Craske, 1998). Indeed, other types of relevant stimuli have been proposed for behavioral, and clinical research (e.g., Fribbles, as shown by Barry et al., 2014). However, we think that low-level visual cues controllability should be systematically evaluated. Each of the normative variables adds value to the stimuli. Beyond their descriptive value, normative variables can reflect various kinds of cognitive processing and be related to specific brain activities. For instance, objects of different categories are known to activate selective patterns of the brain within the dorsal occipital cortex, the superior temporal sulcus, and the ventral temporal cortex. In another experiment, our stimulus set was used in an MEG experiment to draw a comparison with more traditional localizers, such as grayscale pictures. We found that the OB3D stimuli could indeed activate the Lower Occipital Complex (LOC) (Benmussa et al., 2012). So far, the experiment reported here has not been linked to the web experiment list (http://www.wexlist.net/) mainly because the experiment was limited to a specific sample of participants drawn from the RISC database. We expect that will be the case for the following experiments.

### Integration of O3D objects in virtual reality

VR and interactive video gaming (Bioulac et al., 2013) have emerged as new treatment approaches in therapy and rehabilitation. The key components of VR are diagnosis, therapy, education, and training and the medical record. Video games seem more focused on therapy, rehabilitation, and training.

Both approaches seem to be advantageous because they provide an opportunity to practice activities that are otherwise difficult to do in a clinical environment (e.g., at home), although it can still be administered in traditional therapeutic settings. In the latter case, the main advantages are better control and cost effectiveness. It can provide stimuli for individuals who have difficulty in imaging scenes. It can provide opportunities for those individuals who are too phobic to experience real situations, and it can also generate stimuli of greater magnitude than other more standard techniques such as whole alternative or even fantastic worlds (Riva, 2005).

Furthermore, VR programs benefit from being more interesting and even sometimes enjoyable than traditional therapy tasks. One of the immediate consequences is the higher numbers of repetitions the patients are willing to make. What makes these new tools interesting is their versatility. So far, they have been used in situations as diverse as stroke rehabilitation (Laver et al., 2012), phobia rehabilitation (Parsons and Rizzo, 2008) and may prove useful for Alzheimer disease diagnosis and rehabilitation (Serino and Riva, 2014).

### Future implications of having free data and stimuli for clinical purpose

Clinical psychologists work with all age groups from very young children to older people. In doing so, they work with people with mild, moderate, and severe mental health problems. They also help people suffering from learning disabilities, people with physical and sensory handicaps, brain injury, and even people who have alcohol and other drug problems. In addition, they can treat a wide range of physical health problems. The diversity of these clinical situations benefit from the use of virtual environments. Indeed, there are examples of the use of VR in the field of neuropsychology rehabilitation, in older adult psychology services, and in pediatric services. Their use within learning disabilities services in UK has also been discussed (Serino and Riva, 2014).

VR is at the same time technology, communication interface, and compelling experience. Because of population aging, and global economy uncertainty, free tools, such as tests, software (e.g., NeuroVR 2, Riva et al., 2010), and databases, may be key contributions to lower the overall costs and to encourage the patients to contribute by themselves, in a new way of empowerment (see http://www.patientslikeme.com/). New trends are already emerging in patients' contribution through the internet (Wicks et al., 2014).

## Conclusion

We performed a web-based experiment aiming at normalizing a novel visual stimulus database made of 3D scans of “natural” objects. This kind of stimuli allows a controlled parametric tuning of several stimulus characteristics, as well as a large number of versatile transformations. In addition to classical normalization parameters, including RCJ, we measured a dot threshold estimating the information content needed for recognition and categorization. Overall, the present results are consistent with those reported in the literature with another kind of visual stimuli, indicating this stimulus set is well suited for use in a variety of experiments, with healthy subjects or patients.

In addition to the usual normalization data available with other image sets, the possibility to measure a recognition threshold, in terms of the numbers of dots, offers a quantitative evaluation of recognition performance, a feature rarely available with other stimulus sets.

To conclude, in this paper, we have shown that a web-based experiment is well suited to normalize a database aimed at providing visual stimuli (natural objects) for the research community. In addition, such normalization is especially important for clinical research, because the patients can have limited abilities to recognize some objects or some categories.

## Author contributions

Conceived and designed the experiment: Stéphane Buffat, Jean Lorenceau. Scanned the objects: Frédéric Benmussa. Performed the experiment: Delphine Rider. Analyzed the data: Stéphane Buffat, Véronique Chastres. Contributed reagents/material/analysis tools: Alain Bichot, Véronique Chastres. Wrote the paper: Stéphane Buffat, Jean Lorenceau. Funded the project: Jean Lorenceau.

### Conflict of interest statement

The authors declare that the research was conducted in the absence of any commercial or financial relationships that could be construed as a potential conflict of interest.
