# Supplementary material for: OB3D, a new set of 3D objects available for research: a web-based study
Source: Front Psychol. 2014 Oct 6;5:1062. doi: 10.3389/fpsyg.2014.01062 (PMC4186308; doi:10.3389/fpsyg.2014.01062)
Supplement: Supplementary file 2 [file DataSheet1.DOCX]

**Supplementary material**

Supplementary video 1: Example of morphing between objects.

Supplementary Figure 1: H_name_ and Name agreement for all the objects.

Supplementary Table 1: This Table presents the normalization data for all the objects.


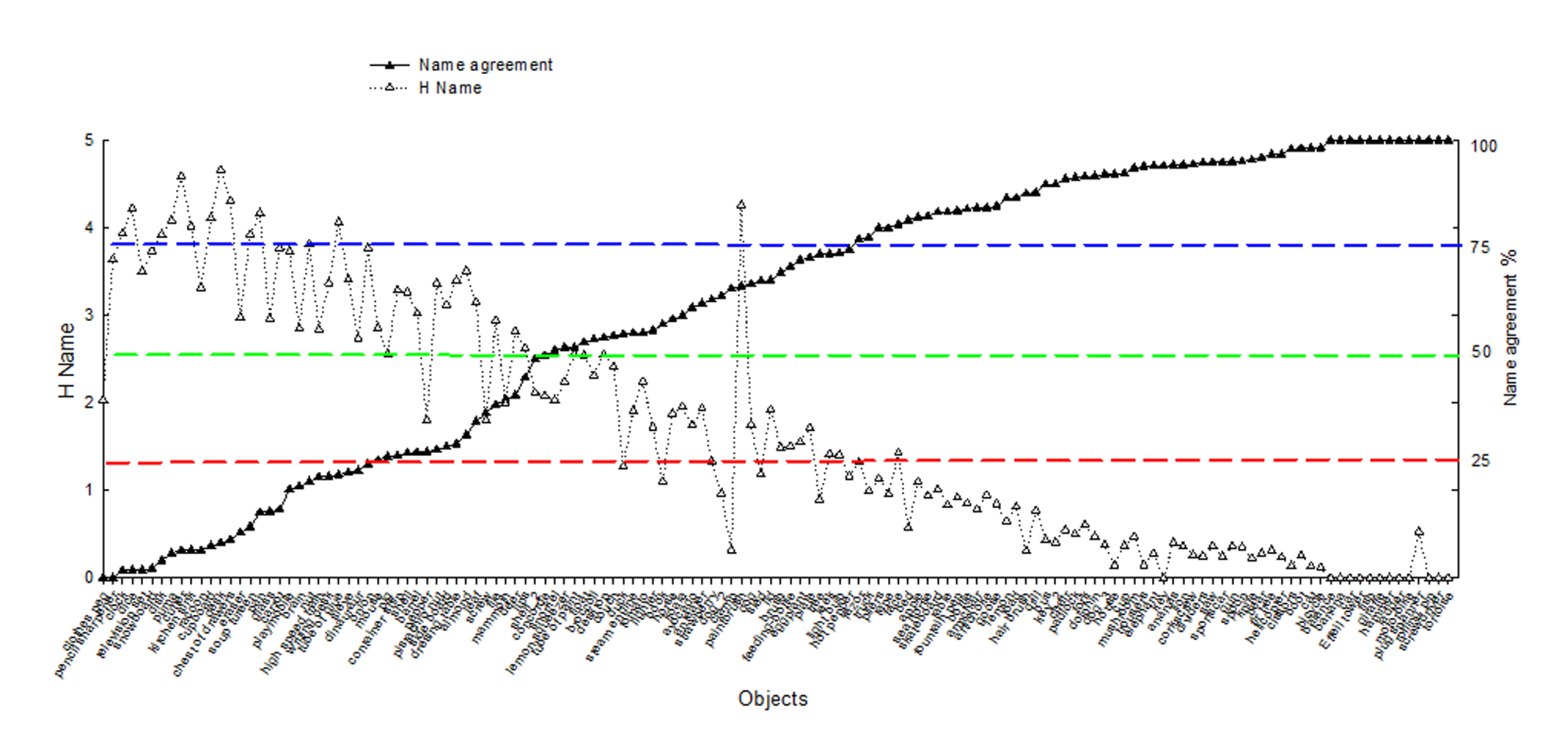


Supplementary Figure 1

| Object | | Name agreement | | Familiarity | Retrospective Confidence Judgment | Number of points | H_name_ | Category  agreement | Number of answers |
| --- | --- | --- | --- | --- | --- | --- | --- | --- | --- |
| almond | | 0.327 | | 4.173 | 3.038 | 6632 | 3.511 | 0.538 | 52 |
| pineapple | | 0.943 | | 7.113 | 7.151 | 4380 | 0.404 | 0.906 | 54 |
| anteater | | 0.627 | | 2.627 | 4.941 | 4472 | 1.936 | 0.941 | 51 |
| apple | | 0.824 | | 8.039 | 7.49 | 2407 | 1.099 | 0.98 | 51 |
| armchair | | 0.846 | | 7.692 | 7.846 | 2550 | 0.783 | 0.942 | 52 |
| artichoke | | 0.846 | | 6.827 | 6.654 | 2153 | 0.951 | 0.981 | 52 |
| avocado | | 0.618 | | 6.6 | 5.527 | 5326 | 1.746 | 0.891 | 55 |
| balance | | 1 | | 5.52 | 7.26 | 2399 | 0 | 0.18 | 50 |
| banana | | 1 | | 7.839 | 8.179 | 1263 | 0 | 0.964 | 56 |
| barrel | | 0.288 | | 4.085 | 3.203 | 12918 | 3.022 | 0.424 | 59 |
| bear | | 0.96 | | 4.54 | 7.4 | 2039 | 0.282 | 0.94 | 50 |
| bed | | 0.818 | | 6.418 | 4.782 | 5718 | 0.571 | 0.818 | 55 |
| bicycle | | 0.983 | | 7.4 | 8.183 | 1339 | 0.122 | 0.9 | 61 |
| book | | 0.58 | | 4.76 | 3.06 | 10571 | 1.104 | 0.62 | 51 |
| boot | | 0.982 | | 7.123 | 7.895 | 1243 | 0.254 | 0.877 | 57 |
| bottle | | 0.843 | | 6.569 | 6.137 | 3493 | 0.853 | 0.412 | 51 |
| brain | | 0.209 | | 2.14 | 1.535 | 10781 | 2.853 | 0.349 | 43 |
| broccoli | | 0.545 | | 6.727 | 6.255 | 4118 | 2.315 | 0.964 | 56 |
| brush | | 0.698 | | 5.603 | 6.444 | 2402 | 1.492 | 0.413 | 63 |
| bus | | 0.9 | | 5.3 | 6.44 | 5989 | 0.436 | 0.9 | 50 |
| car | | 1 | | 7.959 | 7.755 | 2002 | 0 | 0.98 | 49 |
| carrot | | 0.156 | | 4.067 | 2.778 | 6877 | 3.771 | 0.378 | 45 |
| cat | | 0.982 | | 7.364 | 7.8 | 1482 | 0.131 | 0.964 | 55 |
| chair | | 0.912 | | 7.912 | 7.456 | 1941 | 0.549 | 0.86 | 57 |
| chest of drawer | | 0.087 | | 3.826 | 2.043 | 13129 | 4.307 | 0.413 | 46 |
| clamp | | 0.661 | | 4.452 | 6.839 | 2545 | 0.318 | 0.887 | 62 |
| clock | | 0.018 | | 3.281 | 2.509 | 8656 | 3.939 | 0.333 | 57 |
| clothes peg | | 0.667 | | 6.632 | 6.982 | 3057 | 4.263 | 0.333 | 57 |
| Concorde | | 0.509 | | 5.345 | 7.655 | 1368 | 2.083 | 0.891 | 55 |
| container ship | | 0.279 | | 3.836 | 4.262 | 6165 | 3.288 | 0.836 | 61 |
| corkscrew | | 0.946 | | 7.161 | 8.286 | 1259 | 0.262 | 0.821 | 56 |
| couch | | 0.554 | | 6.625 | 6.893 | 3310 | 2.414 | 0.911 | 56 |
| cup | | 0.925 | | 6.962 | 6.358 | 3590 | 0.371 | 0.792 | 53 |
| cupboard | | 0.073 | | 3.509 | 2 | 13267 | 4.114 | 0.291 | 55 |
| cutter | | 0.063 | | 2.891 | 2.484 | 5987 | 4.593 | 0.234 | 64 |
| deer | | 0.418 | | 4.145 | 5.436 | 5277 | 2.819 | 0.927 | 55 |
| desk | | 0.081 | | 5.258 | 4.919 | 5299 | 4.665 | 0.839 | 62 |
| desktop | | 0.549 | | 5.157 | 4.667 | 6763 | 2.554 | 0.588 | 51 |
| dice | | 0.018 | | 1.291 | 1 | 3460 | 4.222 | 0.345 | 55 |
| dinosaur | | 0.245 | | 3.226 | 3.755 | 6887 | 2.737 | 0.868 | 53 |
| disk | | 0.04 | | 4.24 | 3.18 | 7544 | 3.925 | 0.08 | 50 |
| dividers | | 0.949 | | 6.559 | 7.831 | 1008 | 0.251 | 0.695 | 59 |
| dog | | 0.672 | | 5.475 | 5.115 | 5578 | 1.751 | 0.934 | 61 |
| dog_2 | | 0.644 | | 4.511 | 3.622 | 5237 | 0.968 | 0.756 | 45 |
| dog_3 | | 0.923 | | 7.058 | 7.577 | 1827 | 0.377 | 0.962 | 52 |
| dolphin | | 0.918 | | 5.408 | 8.102 | 1444 | 0.474 | 0.98 | 49 |
| dressing table | | 0.306 | | 3.796 | 3.531 | 8522 | 3.4 | 0.694 | 49 |
| drill | | 0.881 | | 6.136 | 7.508 | 1314 | 0.773 | 0.746 | 60 |
| duck | | 0.557 | | 2.475 | 2.623 | 15389 | 1.274 | 0.443 | 61 |
| Object | | | Name agreement | Familiarity | Retrospective Confidence Judgment | Number of points | H_name_ | Category  agreement | Number of answers |
| Eiffel tower | | 1 | | 7.12 | 7.96 | 1177 | 0 | 0.95 | 60 |
| eggplant | | 0.727 | | 7.018 | 6.491 | 2400 | 1.554 | 0.891 | 56 |
| elephant | | 0.942 | | 5 | 6.885 | 3921 | 0.278 | 0.962 | 52 |
| eraser | | 0.104 | | 1.333 | 1.146 | 8175 | 2.978 | 0.042 | 48 |
| face | | 0.807 | | 6.667 | 6.018 | 5015 | 1.427 | 0.702 | 57 |
| feeding-bottle | | 0.712 | | 5.827 | 6.942 | 3310 | 1.508 | 0.673 | 52 |
| file | | 0.396 | | 4.708 | 6.208 | 3848 | 2.945 | 0.917 | 49 |
| fir tree | | 0.968 | | 7.206 | 7.905 | 1327 | 0.316 | 0.968 | 64 |
| fish | | 1 | | 7 | 7.764 | 1804 | 0 | 0.891 | 55 |
| float | | 0.538 | | 4.058 | 5.5 | 3266 | 2.547 | 0.385 | 53 |
| fork | | 0.917 | | 8.467 | 8.267 | 972 | 0.607 | 0.917 | 60 |
| fountain pen | | 0.839 | | 6.464 | 6.321 | 2791 | 0.92 | 0.429 | 56 |
| giraffe | | 1 | | 5.154 | 8.327 | 839 | 0 | 0.981 | 52 |
| glass | | 0.152 | | 6.696 | 5.717 | 3571 | 2.964 | 0.413 | 47 |
| goose | | 0.849 | | 5.415 | 7.415 | 1054 | 0.846 | 0.981 | 53 |
| gun | | 0.951 | | 3.033 | 6.934 | 1997 | 0.361 | 0.689 | 61 |
| hair brush | | 0.878 | | 7 | 6.265 | 4665 | 0.307 | 0.327 | 49 |
| hammer | | 1 | | 7.226 | 8.547 | 834 | 0 | 0.962 | 53 |
| helicopter | | 0.968 | | 4.726 | 7.839 | 1517 | 0.238 | 0.919 | 62 |
| high speed train | | 0.231 | | 2.462 | 2.135 | 8286 | 2.843 | 0.269 | 53 |
| horse | | 0.591 | | 6.386 | 7.864 | 1552 | 1.88 | 1 | 44 |
| hot pepper | | 0.75 | | 5.857 | 6.393 | 2414 | 1.161 | 0.964 | 56 |
| house | | 0.923 | | 6.962 | 5.077 | 8747 | 0.144 | 0.865 | 52 |
| key | | 0.942 | | 7.654 | 8.038 | 2228 | 0 | 0.483 | 58 |
| key_2 | | 0.9 | | 5.9 | 7.78 | 1736 | 0.41 | 0.558 | 52 |
| kitchen sink | | 0.063 | | 2.979 | 3.104 | 10916 | 4.018 | 0.625 | 48 |
| knife | | 0.955 | | 8.212 | 7.697 | 1466 | 0.229 | 0.939 | 66 |
| koala | | 0.6 | | 4.822 | 4.933 | 7902 | 1.955 | 0.844 | 45 |
| ladder | | 1 | | 7.19 | 8.328 | 809 | 0 | 0.672 | 58 |
| leaf | | 0.358 | | 4.472 | 2.943 | 11808 | 3.154 | 0.547 | 53 |
| leek | | 0.741 | | 6.685 | 6.704 | 2159 | 1.419 | 0.889 | 54 |
| lemon | | 0.868 | | 6.925 | 6.302 | 3840 | 0.651 | 0.906 | 53 |
| lemon squeezer | | 0.527 | | 5.109 | 4.8 | 6228 | 2.244 | 0.545 | 55 |
| lid | | 0.68 | | 6.22 | 5.94 | 4661 | 1.921 | 0.64 | 50 |
| light bulb | | 0.742 | | 6.097 | 5.274 | 7056 | 1.408 | 0.565 | 63 |
| lighter | | 0.565 | | 4.522 | 4.935 | 8378 | 1.72 | 0.174 | 46 |
| lion | | 0.944 | | 4.5 | 7.444 | 2089 | 0.361 | 0.981 | 54 |
| lips | | 0.458 | | 4.78 | 3.576 | 6456 | 2.629 | 0.593 | 59 |
| mammoth | | 0.408 | | 4.694 | 7.776 | 1637 | 1.991 | 0.98 | 49 |
| motorbike | | 1 | | 6.373 | 7.961 | 1929 | 0 | 0.961 | 51 |
| mouse | | 0.269 | | 4.942 | 6.654 | 4787 | 2.859 | 0.962 | 52 |
| mushroom | | 0.937 | | 6.063 | 5.952 | 3977 | 0.474 | 0.921 | 63 |
| nut | | 0.22 | | 4.92 | 3.44 | 9212 | 3.814 | 0.72 | 50 |
| onion | | 0.26 | | 4.48 | 3.02 | 9456 | 3.771 | 0.5 | 52 |
| padlock | | 0.915 | | 5.864 | 6.661 | 3136 | 0.5 | 0.373 | 59 |
| paintbrush | | 0 | | 1.25 | 1.231 | 7840 | 2.033 | 0.096 | 52 |
| pan | | 0.15 | | 5.617 | 5.383 | 3160 | 4.171 | 0.567 | 60 |
| pencil sharpener | | 0 | | 1.039 | 0.608 | 8205 | 3.64 | 0.02 | 51 |
| pepper | | 0.288 | | 7.135 | 5.904 | 3817 | 1.806 | 0.904 | 52 |
| pig | 0.278 | | | 2.833 | 2.741 | 10177 | 2.562 | 0.704 | 55 |
| Object | Name agreement | | | Familiarity | Retrospective Confidence Judgment | Number of points | H_name_ | Category  agreement | Number of answers |
| pistachio nut | 0.294 | | | 4.078 | 3.235 | 10541 | 3.369 | 0.451 | 51 |
| plate | 0.732 | | | 7.393 | 6.286 | 3362 | 1.715 | 0.768 | 56 |
| playmobil | 0.203 | | | 6.831 | 6.814 | 2780 | 3.736 | 0.746 | 59 |
| pliers | 0.8 | | | 6.16 | 7.18 | 1782 | 1.132 | 0.94 | 52 |
| plug spanner | 1 | | | 8.086 | 8.448 | 1043 | 0.53 | 0.96 | 51 |
| puma | 0.057 | | | 4.792 | 7.057 | 1368 | 4.089 | 0.981 | 53 |
| raccoon | 0.063 | | | 5.083 | 5.604 | 3645 | 3.315 | 0.979 | 48 |
| razor | 0.774 | | | 6.868 | 7.434 | 1874 | 1.322 | 0.226 | 53 |
| rolling pin | 1 | | | 6.674 | 7.739 | 964 | 0 | 0.957 | 46 |
| saw | 0.95 | | | 6.183 | 7.417 | 3108 | 0.366 | 0.933 | 60 |
| scissors | 0.98 | | | 8.216 | 8.706 | 662 | 0.139 | 0.529 | 51 |
| screw | 0.377 | | | 6.755 | 7.491 | 2184 | 1.809 | 0.679 | 54 |
| screwdriver | 1 | | | 7.033 | 7.733 | 1045 | 0 | 0.94 | 50 |
| sea horse | 0.827 | | | 3.846 | 7.635 | 1647 | 0.937 | 0.942 | 52 |
| shell | 0.286 | | | 3.306 | 3.02 | 13357 | 3.263 | 0.592 | 49 |
| shell_2 | 0.5 | | | 4.214 | 4.571 | 4346 | 2.118 | 0.548 | 42 |
| side board | 0.3 | | | 5.6 | 5.22 | 6117 | 3.123 | 0.92 | 50 |
| sieve | 0.241 | | | 5.914 | 4.569 | 4813 | 3.408 | 0.793 | 58 |
| skateboard | 0.836 | | | 5.273 | 7.382 | 1660. | 1.013 | 0.545 | 55 |
| sled | 0.679 | | | 3.589 | 4.464 | 6068 | 1.188 | 0.464 | 56 |
| snorkel | 0.52 | | | 3.28 | 4.38 | 4817 | 2.032 | 0.34 | 50 |
| snowboard | 0.022 | | | 2.761 | 3.152 | 6031 | 3.735 | 0.217 | 46 |
| soup tureen | 0.117 | | | 6.317 | 5.9 | 3853 | 3.926 | 0.933 | 60 |
| spoon | 0.952 | | | 8.095 | 7.841 | 1168 | 0.352 | 0.905 | 63 |
| sports car | 0.95 | | | 6.817 | 6.467 | 2813 | 0.247 | 0.95 | 60 |
| steam engine | 0.56 | | | 4.14 | 4.7 | 8778 | 1.91 | 0.68 | 50 |
| strawberry | 0.636 | | | 5.582 | 5.055 | 6862 | 1.334 | 0.655 | 55 |
| table | 0.836 | | | 7.098 | 6.148 | 2926 | 0.836 | 0.836 | 61 |
| tape | 0.8 | | | 5.444 | 5.156 | 8199 | 0.961 | 0.356 | 45 |
| television set | 0.018 | | | 1.614 | 1.421 | 12772 | 3.503 | 0.368 | 58 |
| toad | 0.868 | | | 5.509 | 5.981 | 3233 | 0.814 | 0.925 | 53 |
| tomato | 0.56 | | | 6.76 | 5.52 | 5602 | 2.245 | 0.9 | 50 |
| tortoise | 1 | | | 5.566 | 7.547 | 1421 | 0 | 0.981 | 53 |
| tree | 0.74 | | | 5.82 | 5.06 | 5072 | 0.897 | 0.82 | 50 |
| trousers | 0.94 | | | 7.7 | 6.04 | 4675 | 0.146 | 0.96 | 51 |
| truck | 0.778 | | | 4.315 | 4.63 | 6599 | 0.993 | 0.852 | 54 |
| tube of glue | 0.236 | | | 2.891 | 2.545 | 7292 | 4.067 | 0.055 | 55 |
| tube of paint | 0.527 | | | 4.655 | 4.691 | 4540 | 2.551 | 0.091 | 55 |
| writing desk | 0.232 | | | 2.429 | 2.5 | 9914 | 3.372 | 0.411 | 56 |

Supplementary Table 1: Normalization data for all objects
